# Three-Year Follow-up of PositiveLinks: Higher Use of mHealth Platform Associated with Sustained HIV Suppression

**DOI:** 10.1007/s10461-024-04405-z

**Published:** 2024-06-13

**Authors:** Catherine Bielick, Chelsea Canan, Karen Ingersoll, Ava Lena Waldman, Jason Schwendinger, Rebecca Dillingham

**Affiliations:** 1 Division of Infectious Diseases and International Health, University of Virginia, Charlottesville, VA USA; 2 Division of Disease Prevention, Virginia Department of Health, Richmond, VA USA; 3 Department of Psychiatry and Neurobehavioral Sciences, University of Virginia, Charlottesville, VA USA

**Keywords:** HIV, Medication adherence, AIDS, Patient care, Antiretroviral

## Abstract

PositiveLinks (PL) is a mHealth platform to support care engagement by people with HIV (PWH). Daily reminders prompt the user to report medication adherence, mood, and daily stress. Higher response rate to PL check-ins has been associated with better suppression of viral load over 6–18 months. We conducted a retrospective chart review for a three-year period collecting demographic information, average mood and stress scores, and all viral loads obtained in usual patient care. We performed multivariable logistic regression modeling to identify factors associated with loss of viral load suppression and a time-to-event survival analysis until first unsuppressed viral load stratified by PL usage. Of the 513 PWH included, 103 had at least one episode of viral non-suppression. Low users of PL were more likely to have an unsuppressed viral load with an adjusted Odds Ratio (aOR) of 5.8 (95% CI 3.0-11.5, *p* < 0.001). Protective factors included older age (aOR 0.96; 95% CI 0.93–0.98, *p* = 0.003) and income above the federal poverty level (FPL) (aOR 0.996; 95% CI 0.995–0.998, *p* < 0.001). High PL use was also associated with better viral load suppression (VLS) over time (*p* < 0.0001 ((aHR) of 0.437 (95% CI 0.290–0.658, *p* < 0.001)) after adjusting for age and FPL. High stress scores were related to subsequent loss of viral suppression in an exploratory analysis. High check-in response rate on the PL app, older age, and higher income are associated with sustained VLS over time. Conversely, lack of response to check-ins or increased reported stress may signal a need for additional support.

## Introduction

People living with HIV (PWH) require ongoing and uninterrupted treatment to maintain viral load suppression (VLS) over time [1–3]. Unsuppressed viral loads can cause damage to the immune system leading to infection with a deadly opportunistic infection, transmission of HIV to other people, or even death [4–6]. A recent estimate from the CDC showed that 34.1% of PWH in the United States who are engaged in care had not yet achieved viral load suppression in 2021 and 18.1% had no adequate linkage to care within one month of diagnosis [7]. HIV is now considered a chronic disease requiring, in most cases, daily medications. Therefore, one primary aim of HIV care is to support the millions of people who currently live with HIV to engage consistently with care [8–10]. To do so we must develop and scale innovative, effective, and affordable tools which can support each person in an individualized way [11].

For longer than twenty years mobile health technology (mHealth) has supported chronic disease management through methods including short message service (SMS) reminders, telephone counseling, smartphone diaries, and advanced symptom management systems [11–17]. PositiveLinks (PL) is an mHealth platform for engagement with care for PWH and is offered as usual care at the Ryan White HIV/AIDS Program (RWHAP)-funded Clinic (RWC) in our academic institution [18–28]. Consistent with Information-Motivation-Behavioral Skills Model (IMB) and Social Action Theories [11], self-monitoring is a key activity on PL [18, 19, 21]. Daily check-ins prompt the user to report medication adherence as well as mood and stress levels, which are well-described barriers to optimal care engagement for PWH [29]. Consistent with the IMB model, users can also review calendar-based displays of their check-in data over time, receiving feedback. Response rate to all forms of check-ins on PL has been associated with better suppression of viral load over 6–18 months [19], and the treatment cohort has grown since our previous reports. In this study, we evaluate, in a large cohort, the relationship between completion of PL-delivered check-ins with maintenance of viral load suppression over three years of follow-up from the date of PL enrollment. We have previously defined high or low usage of the application (app) based upon a threshold of 48% overall mean response rate to check-ins. We hypothesized that among patients with a high response rate to PL daily check-ins (≥ 48%), we would observe a higher degree of VLS than among patients with low usage of PL.

## Materials and Methods

### Study Design, Population, and Data Sources

This was a retrospective cohort study in which data collection began after a run-in period of one-month or six months (varies by analysis, discussed in statistical analysis section) after enrollment in the PL app to ensure exposure to the PL intervention. This retrospective study was approved by the University of Virginia Institutional Review Board as exempt with a waiver of informed consent. This sample included people with HIV who were suppressed at baseline and were receiving care in the RWC at the University of Virginia Medical Center who first enrolled in the PL app between July 1, 2017 and December 31, 2020. The RWC serves patients who live up to several hundred miles away from the clinic, including many who live in rural areas. All participants were 18 years of age or older and had been diagnosed with HIV. Because participants had to have at least six months of follow-up for this analysis, the data collection period ended on June 30, 2021. Additionally, included patients had at least one viral load checked during the period from 1 month following enrollment in PL to either 36 months after PL enrollment or June 30, 2021, whichever came first. A participant may have been deactivated from PL at any time (“deactivation date”) by the patient’s own wishes, discharge from the clinic, or patient death at which time data collection ceased.

PL usage data is stored by patient identification number and contains the following metrics: time stamp and type of check-in submitted, time of private message sent, time of a community message board post, any HIV viral load obtained as a part of usual care, and patient demographic information. Viral load measurements were restricted to the data collection window and transformed to a binary value; levels greater than or equal to 200 copies/mL were designated as “unsuppressed” and those with less than 200 copies/mL were coded as “suppressed.”

### Intervention

**Fig. 1:**
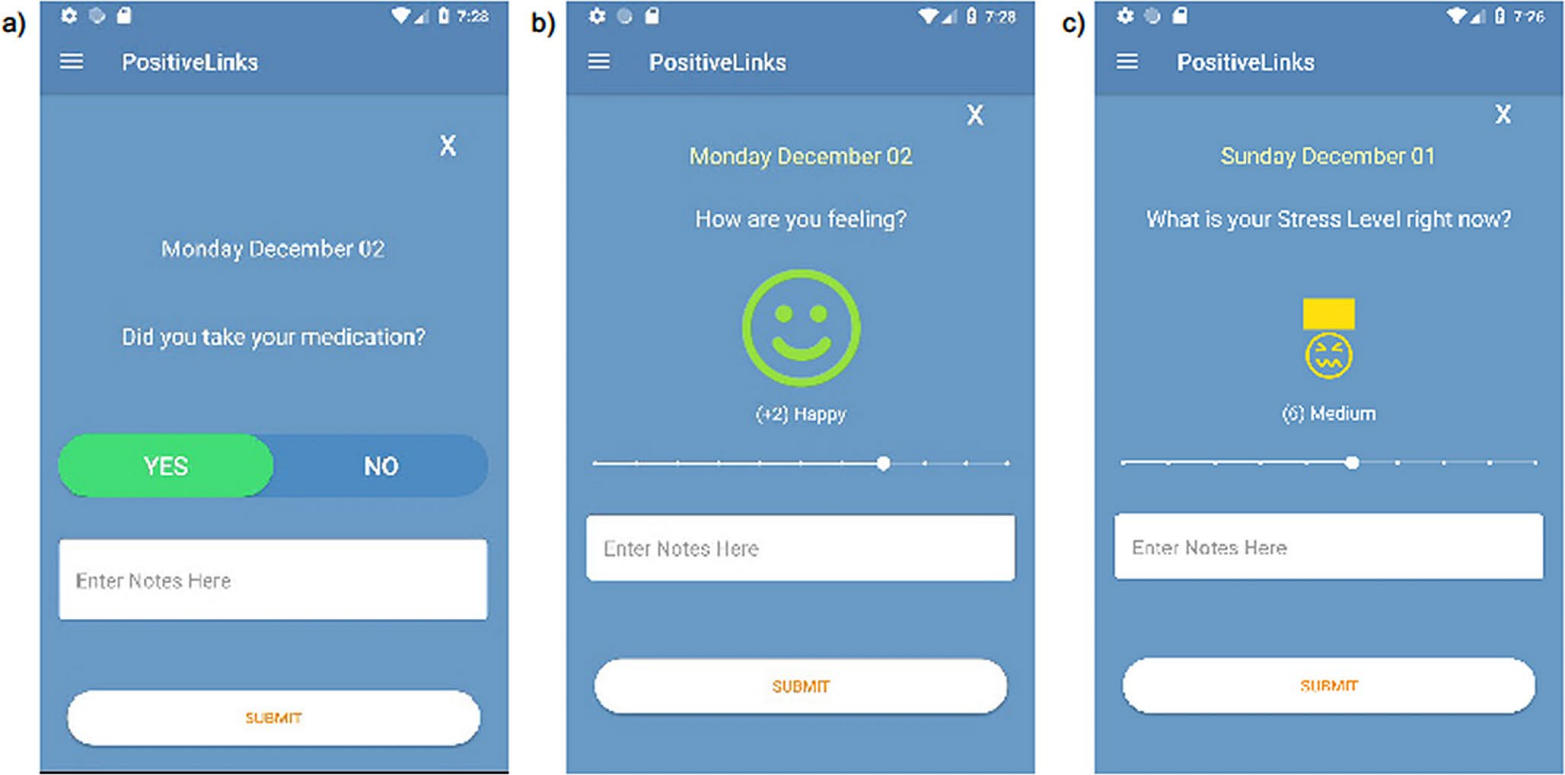
Example of daily check-ins: (**a**) medication adherence, (**b**) mood level, (**c**) stress level

PL was the intervention for our study. During the period of this study, all patients in the RWC were offered enrollment in the PL app as usual care. Because the app is no longer in its pilot phase, patients who declined enrollment were not followed or quantified. Upon enrollment, PL is installed on patients’ own smartphone device. If patients do not have a smartphone, they are given a pre-paid smartphone on which the PL app is installed. At enrollment, PL team members provide training on how to use a smartphone and the PL app. Previous studies have reported the effectiveness of PL in its research (version 1.x) and pilot (2.x) periods [18, 19, 21]. This study reports on outcomes when PL was offered as usual care in its PL 3.x version. The full range of features the app offers now includes: (1) daily self-monitoring check-ins, (2) a private community board for patient users to communicate anonymously with each other, (3) private and secure messaging between the PL member and their care team, (4) tracking of HIV viral load and CD4 lab values over time, (5) educational resources and Frequently Asked Questions (FAQs) that address questions and barriers to living well with HIV, 5) weekly quizzes, (6) secure document upload, (7) telehealth, (8) appointment reminders, and (9) automated report generation summarizing patient engagement with the platform for administrators. For their daily check-ins, the member submits a “yes/no” response to the medication daily check-in and ratings of their current mood (scale − 5 to 5) and stress level (scale 0 to 10) (Fig. 1). A higher mood score is interpreted as feeling more positive and a higher stress score is feeling more distress. Features unique to PL 3.0 are telehealth video calling through direct messaging links, improved accessibility of telehealth by turning on the camera and microphone by default, the ability to upload documents electronically, and automated reporting on patient engagement.

### Outcomes

The primary outcome for this analysis was to identify sociodemographic and PL usage characteristics associated with developing an unsuppressed viral load over time. Because PL usage can change over time, we conducted a secondary analysis to examine the association between PL usage and viral suppression using independent 6-month blocks of time, where the PL usage category could change with each 6-month period. In this secondary analysis, the outcome was the percentage of PL members maintaining viral suppression for the full 6 months following their enrollment date. Lastly, in an exploratory analysis, we looked at median stress scores immediately preceding a loss of viral suppression for every member who had a viral load conversion to unsuppressed.

### Statistical Analysis

All members in the enrollment period with at least 6 months of data were separated into the following groups: high PL usage (≥ 48% check-in response rate) or low PL usage (< 48% check-in response rate). For time-to-event analysis PL usage was measured in the period prior to the first viral load becoming unsuppressed or overall if the person did not become unsuppressed. For non-time series analyses, usage was calculated across the entire monitoring period for all members. The monitoring period was from the enrollment date until the earliest of the following dates: deactivation date, 36 months from enrollment date, or the predefined end date of June 30, 2021. Viral loads and check-in response rates were collected within those intervals after a one-month run-in period. Demographic characteristics included in the analysis were age, sex, race/ethnicity, insurance type, income as a percentage of FPL, years from HIV diagnosis to PL enrollment, and mean mood and stress scores across the entire observation period. Differences between groups were assessed using Chi Square tests or Fisher’s exact tests on categorical variables and unpaired t-tests or Mann Whitney Wilcoxon rank sum tests on continuous variables as appropriate. We used a logistic regression model to estimate the association between PL usage during the entire monitoring interval and viral suppression. We controlled for the following potential confounding factors in this model: age, sex, race/ethnicity, FPL, and time between HIV diagnosis and enrollment.

We used a Kaplan-Meier survival curve and Cox proportional hazards model to evaluate the probability of a participant with a suppressed viral load at baseline developing an unsuppressed viral load during the study period. An unsuppressed viral load at baseline status was defined as at least one unsuppressed viral load between the 6 months preceding enrollment until 1 month after enrollment; a suppressed viral load at baseline status required confirmed suppressed viral loads in that interval. We stratified by PL usage group as measured only prior to the initial viral load conversion or any censoring event either due to deactivation or reaching the end date of monitoring (June 30, 2021). Only covariates with a *p*-value < 0.05 were included in the multivariable analysis. We also compared the percentage of members with suppressed viral loads between PL usage groups for each 6-month time interval after enrollment using a Fisher’s exact test. Because the first six months after a member’s enrollment included the one-month run-in period and to best replicate methods of prior PL studies, we started this analysis at month six after enrollment. Members could cross between PL usage groups or suppression groups at different time periods independent from their initial grouping.

Lastly, we tested for significant differences between median stress score distributions between the three separate months preceding viral load conversion events using the Kruskal-Wallis H test. As a comparator, we ran the same analysis on stress scores among members who did not experience loss of VLS. To control for timing of world events as confounders to stress scores, the comparison group was selected based on enrollment date, whereby each member with loss of VLS was paired with the next sequentially enrolled member who did not experience a loss VLS. Stress scores in the comparison group were measured at the same number of months post-enrollment as their unsuppressed counterparts. All data were analyzed using R version 4.3.0 and R Studio (R Foundation for Statistical Computing).

## Results

### Demographic and Application Usage Characteristics

There were 630 PL member accounts with data between the study dates of July 1, 2017 and June 30, 2021. This is an average of 77.4% of the total number of patients in the Ryan White Clinic per year during this time period. There were 111 profiles excluded because they did not have at least 6 months of data after enrollment and 6 test or incomplete profiles, leaving 513 total profiles. There were 392 profiles categorized as “high users” and 121 as “low users.” The median age of the entire sample was 46 (IQR 35–56) years-old, with 24.9% women, 73.1% men, and 1.9% transgender women participants (Table 1). The sample was White (49.1%), Black or African-American (42.5%), with 1.8% reporting more than one race. Regarding ethnicity, 5.1% reported they were Hispanic. The median FPL for the entire sample was 105% (22 – 243%), where among high users the median was 115% (49-244%) and 76% (0-211%) among low users (W = 27,377; *p* = 0.01). High users had a higher proportion of Medicare Part A/B insurance (24% compared with 13% of low users) and a higher median number of months from enrollment to any viral load (11.5, 7.5–15.0) compared with low users (7.0 (5.0–13.0)). Low users had a higher proportion of people without health insurance (12% compared with 3.9% of high users) (χ^2^ = 13; *p* = 0.011). High users also reported lower median stress score (3.5, 2.2-5.0) and higher mood score (0.5, (-0.1)-1.9) compared with low users’ median scores which were 4.0 (2.4-5.0) and 0.4 ((-0.2)-1.5) respectively, though these differences were not statistically significant.

**Table 1:** Demographic and application usage characteristics by usage group

Variable	Total, *N* = 513^1^	Low Users^1^, *n* = 121 (23.6%)	High Users^1^, *n* = 392 (76.4%)	Test Statistic^2^	*p*-value^2^
**Age**	46 (35–56)	41 (30, 51)	48 (37, 57)	30,120	< 0.001
**Sex**				NA	0.7
Female	375 (73.1%)	86 (71.1%)	289 (73.7%)		
Male	128 (24.9%)	32 (26.4%)	96 (24.5%)		
Transgender Woman	10 (1.9%)	3 (2.5%)	7 (1.8%)		
**Race**				NA	0.091
White (non-Hispanic)	252 (49.1%)	49 (40.5%)	204 (52.0%)		
Asian	7 (1.4%)	2 (1.7%)	5 (1.3%)		
Black or African-American	218 (42.5%)	58 (47.9%)	160 (40.8%)		
Hispanic	26 (5.1%)	10 (8.3%)	16 (4.1%)		
More than one race	9 (1.8%)	2 (1.7%)	7 (1.8%)		
**FPL**	105 (22–243)	76 (0-211)	115 (49–244)	27,377	0.010
**Years between HIV Diagnosis to Enrollment, median**	10.2 (2.7–17.3)	8 (3–15)	11 (3–18)	24,292	0.2
**Insurance**				13	0.011
Medicaid	172 (33.5%)	38 (31.5%)	134 (34.2%)		
Medicare Part A/B	92 (17.9%)	12 (9.9%)	80 (20.4%)		
No Insurance	24 (4.7%)	11 (9.1%)	13 (3.3%)		
Private - Employer	91 (17.7%)	20 (16.5%)	71 (18.1%)		
Private - Individual	46 (9.0%)	8 (6.6%)	38 (9.7%)		
Missing	88 (17.2%)	32 (26.4%)	56 (14.3%)		
**Baseline Viral Load**				0.82	0.4
Suppressed at Baseline^3^	328 (63.9%)	73 (60.3%)	255 (65.1%)		
Unsuppressed at Baseline^3^	174 (33.9%)	45 (37.2%)	129 (32.9%)		
Missing	11 (2.2%)	3 (2.5%)	8 (2.0%)		
**Median Number of Months from Enrollment to Any Viral Load Measurement**	10.2 (2.7–17.3)	7.0 (5.0–13.0)	11.5 (7.5–15.0)	29,980	< 0.001
**Number of Viral Load Tests**	4 (2–6)	2 (1–4)	5 (3–6)	33,618	< 0.001
**Stress**	3.7 (2.2-5.0)	4.0 (2.4, 5.0)	3.6 (2.2, 5.0)	21,891	0.3
**Mood**	0.5 (-(0.1)-1.7))	0.4 (-(0.2)-1.5)	0.5 (-(0.1)-1.9)	24,998	0.4

^1^Median (IQR); n (%)

^2^Wilcoxon rank sum test; Fisher’s exact test; Pearson’s Chi-squared test

^3^As measured in the 6 months preceding enrollment, unsuppressed at baseline indicates any detectable viral load during that time.

## Loss of Viral Suppression

Among 513 included participants, there were 103 (20.1%) who were unsuppressed at least once during the study period and 410 (79.9%) who were suppressed throughout the entire period (Table 2). Among the uncensored suppressed group, there were 69 people who were deactivated prior to the end of the monitoring period for any reason of which 24 were high users and 45 were low users. The median number of days to deactivation for high users in the suppressed group was 716 (IQR 476–837) compared with 482 (IQR 329–783) for low users. Age was lower in the unsuppressed group (40, 32–49) than the suppressed group (48, 37–57) with an adjusted odds ratio (aOR) of developing at least one unsuppressed viral load of 0.96 (0.93–0.98; *p* = 0.003) for each additional year of age. The Asian race/ethnicity group had an aOR of 9.40 (1.30–62.5, *p* = 0.02) compared to the white race/ethnicity reference group, but other race and ethnic groups were not significantly different. The percentage of people with income above FPL was lower in the unsuppressed group (58%, 0-149%) than the suppressed group (116%, 53-251%) with an aOR of 0.996 (0.995–0.998; *p* < 0.001) for each additional percentage of income compared with the FPL. The median number of years between HIV diagnosis to enrollment was fewer in the unsuppressed group (7, 1–14) than the suppressed group (11, 3–18) with an aOR of 1.05 (1.01–1.09; *p* = 0.014) for each additional year. The proportion of participants suppressed at baseline was significantly greater among those who were suppressed throughout the entire study period (295 of 410, 72%) compared to those who were unsuppressed at least once during the study period (33 of 103, 32%) (aOR of 8.93 (4.8–17.5; *p* < 0.001)). People who were unsuppressed at least once also had higher average viral load tests performed, 6 (3–8) compared with 4 (2–6) with an increased aOR of 1.37 (1.19–1.59; *p* < 0.001) for each additional blood draw associated with an unsuppressed viral load. Median number of months from enrollment to any measured viral load was higher among the group who became unsuppressed at least once, 13.0 (9.0-17.3) compared with 10.5 (6.5–14.0), with an aOR of 1.16 (1.08–1.24; *p* < 0.001).

**Table 2:** Multivariable analysis of characteristics associated with developing an unsuppressed viral load

	Characteristics of Members by Viral Suppression Status		Odds of Characteristics Associated with Development of Unsuppressed Viral Load			
Variable	Suppressed all of enrollment^1^	Unsuppressed at least once^1^	Unadjusted OR (95% CI)^3^	*p*-value	Adjusted OR (95% CI)^3^	*p*-value
Total	410 (79.9%)	103 (20.1%)				
**PL Usage Group**						
High users	330 (80%)	62 (60%)	**—**		—	
Low users	80 (20%)	41 (40%)	2.58 (1.58–4.19)	< 0.001	5.78 (2.98–11.5)	< 0.001
**Age**	48 (37–57)	40 (32–49)	0.96 (0.94- 0.98)	< 0.001	0.96 (0.93–0.98)	0.003
**Sex**						
Male	306 (74.6%)	69 (67.0%)	—		—	
Female	95 (23.2%)	33 (32.0%)	1.54 (0.95–2.47)	0.08	0.92 (0.49–1.71)	0.8
Transgender Woman	9 (2.2%)	1 (1.0%)	0.49 (0.03–2.70)	0.51	0.14 (0.01–0.97)	0.068
**Race**						
White (non-Hispanic)	211 (51.4%)	41 (39.8%)	—		—	
Asian	5 (1.2%)	2 (1.9%)	2.06 (0.29–9.99)	0.40	9.40 (1.30–62.5)	0.02
Black or African-American	168 (41.0%)	50 (48.5%)	1.53 (0.97–2.44)	0.07	1.86 (0.99–3.53)	0.06
Hispanic	20 (4.9%)	6 (5.8%)	1.54 (0.54–3.90)	0.38	2.49 (0.65–8.89)	0.21
More than one race	6 (1.5%)	3 (2.9%)	2.57 (0.52–10.24)	0.20	0.99 (0.21–4.15)	> 0.9
**FPL**	117 (53–251)	58 (0-149)	0.996 (0.994–0.998)	< 0.001	0.996 (0.995–0.998)	< 0.001
**Years between HIV Diagnosis to Enrollment**	11 (3–18)	7 (1–14)	0.97 (0.95–0.99)	0.02	1.05 (1.01–1.09)	0.014
**Insurance**						
Medicaid	124 (30.2%)	48 (46.6%)	—		—	
Medicare Part A/B	79 (19.3%)	13 (12.6%)	0.43 (0.21–0.97)	0.01	0.88 (0.38–2.03)	0.80
No Insurance	19 (4.6%)	5 (4.9%)	0.68 (0.21–1.81)	0.47	0.34 (0.10–1.02)	0.06
Private - Employer	81 (19.8%)	10 (9.7%)	0.32 (0.14–0.65)	0.002	2.77 (1.04–7.31)	0.04
Private - Individual	38 (9.3%)	8 (7.8%)	0.54 (0.22–1.20)	0.15	0.95 (0.31–2.77)	> 0.90
Missing	69 (16.8%)	19 (18.4%)				
**Baseline Viral Load** ^**4**^						
Suppressed at Baseline	295 (71.9%)	33 (32.0%)	—		—	
Unsuppressed at Baseline	107 (26.1%)	67 (65.1%)	5.60 (3.52–9.07)	< 0.001	8.93 (4.75–17.50)	< 0.001
Missing	8 (2.0%)	3 (2.9%)				
**Number of Viral Load Tests Performed**	4 (2–6)	6 (3–8)	1.31 (1.20–1.44)	< 0.001	1.37 (1.19–1.59)	< 0.001
**Median Months from Enrollment to Any Viral Load**	10.5 (6.5–14.0)	13.0 (9.0-17.2)	1.11 (1.06–1.16)	< 0.001	1.16 (1.08–1.24)	< 0.001
**Stress**	3.7 (2.2, 5.0)	3.7 (2.7, 5.0)	1.08 (0.96–1.23)	0.17	1.09 (0.86–1.38)	0.50
**Mood**	0.5 (-(0.1), 1.8)	0.4 (-0.3), 1.54)	0.90 (0.79–1.02)	0.11	0.86 (0.68–1.09)	0.20

^1^Median (IQR); n (%)

^2^Wilcoxon rank sum test; Fisher’s exact test; Pearson’s Chi-squared test

^3^OR = Odds Ratio, CI = Confidence Interval

^4^As measured in the 6 months preceding enrollment, unsuppressed at baseline indicates any detectable viral load during that time.

**Fig. 2:**
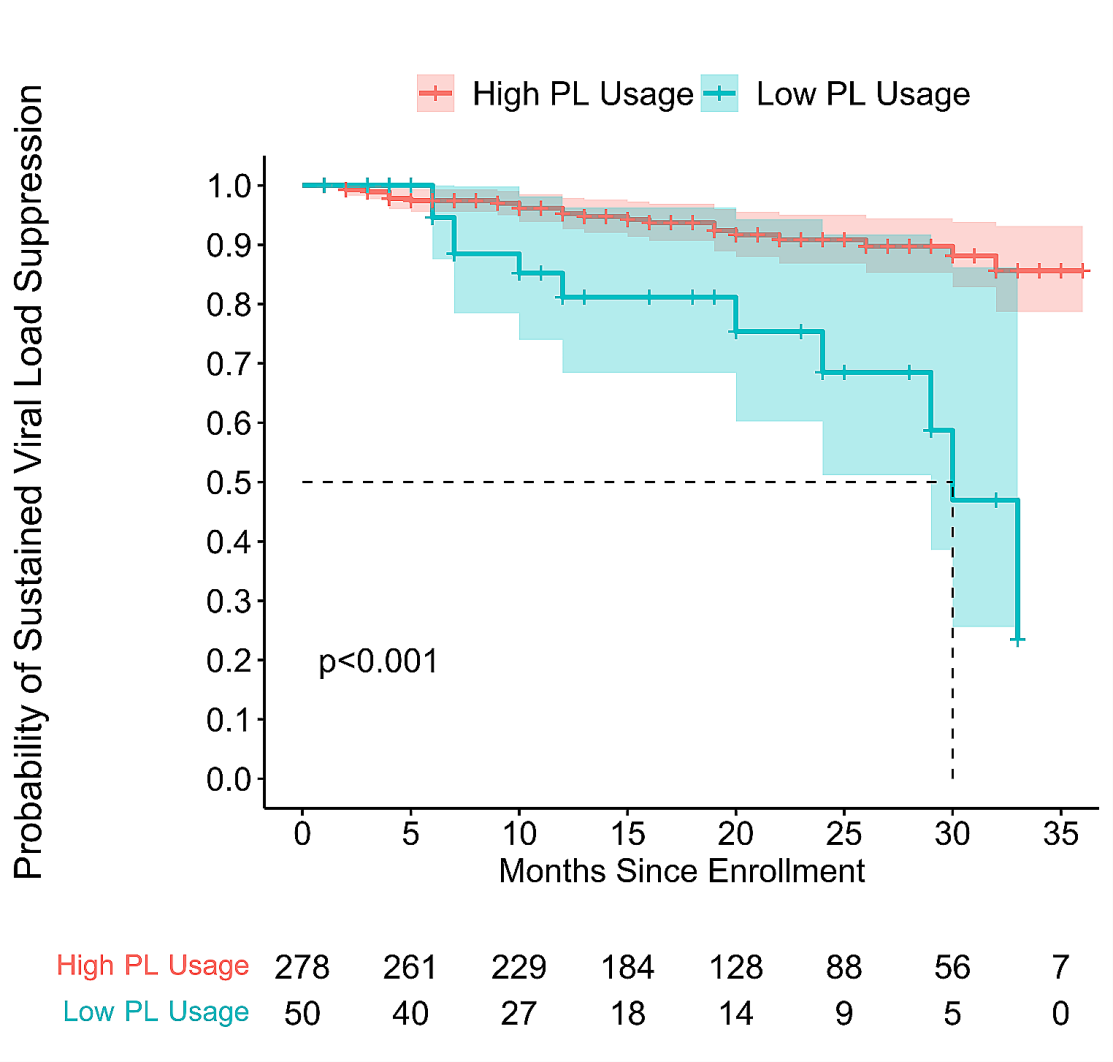
Kaplan-meier curve comparing the probability of maintaining a suppressed viral load between high and low PL users. PL: PositiveLinks

For the time-to-event analysis, there were 328 profiles confirmed to be suppressed at baseline for inclusion in time-to event analysis. In a Kaplan-Meier survival analysis, low PL usage was associated with a higher probability of developing an unsuppressed viral load across 5,985 person-months in the unadjusted survival analysis (*p* < 0.001) with a median time to event of 30 months for low PL users after a one-month run-in period (Fig. 2). In the Cox proportional hazards model, (Table 3) low PL usage was associated with increasing probability of developing an unsuppressed viral load with a univariate hazard ratio of 4.22 (2.04–8.74, *p* < 0.001) and an adjusted hazard ratio of 2.54 (1.16–5.54, *p* = 0.019) when adjusting for age and income compared to FPL. FPL independently conveyed an adjusted hazard ratio of 0.995 (0.991–0.999, *p* = 0.007) for each additional income percentage compared with FPL; age had an adjusted HR of 0.96 (0.93–0.99, *p* = 0.006) for each additional year of age. Self-reported race and sex did not significantly influence sustained viral suppression.

**Table 3:** Cox Proportional Hazards Model, Time until Loss of Viral Suppression

Characteristic	n (%)	HR (univariable)	p-value	HR (multivariable)	p-value
High users	278 (84.8%)	-	-		
Low users	50 (15.2%)	4.22 (2.04–8.74)	< 0.001	2.54 (1.16–5.54)	0.019
**Age**					
Mean (SD)	48.7 (12.4)	0.95 (0.92–0.98)	< 0.001	0.96 (0.93–0.99)	0.006
**Sex**					
Male	238 (72.6%)	-	-		
Female	85 (25.9%)	0.82 (0.37–1.82)	0.633	-	-
Transgender Woman	5 (1.5%)	NA	NA	-	-
**Race**					
White (non-Hispanic)	172 (52.4%)	-	-		
Black or African-American	134 (40.9%)	1.58 (0.77–3.27)	0.211		
Hispanic	17 (5.2%)	2.20 (0.49–9.81)	0.303		
Asian	5 (1.5%)	3.90 (0.50-30.17)	0.192		
**Income compared to FPL**					
Mean (SD)	168.6 (181.8)	0.995 (0.991–0.999)	0.007	0.996 (0.992–0.999)	0.015

HR: Hazard ratio, SD: Standard Deviation, FPL: Federal Poverty Level

### Maintenance of Viral Suppression

**Fig. 3:**
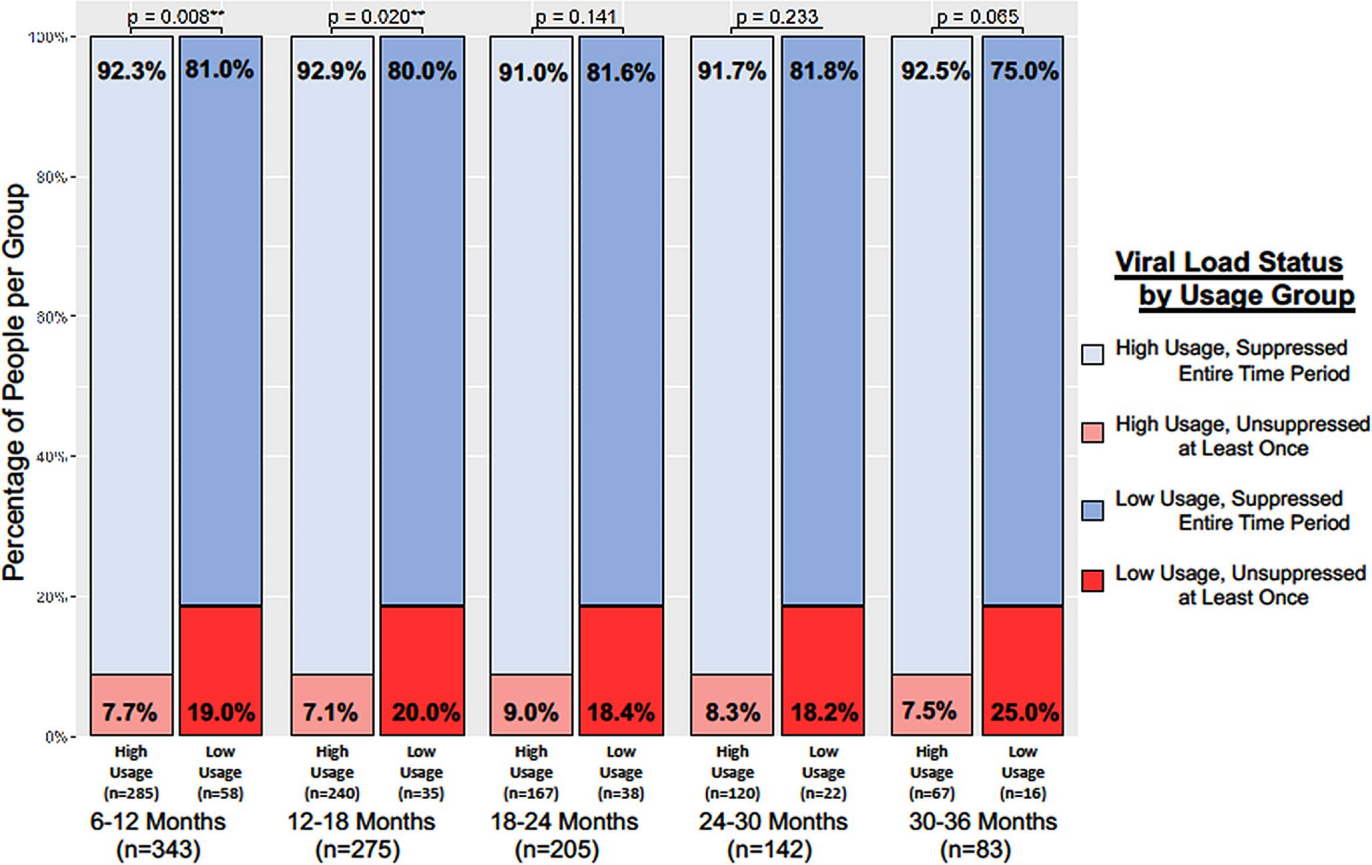
Percentage of people with viral load suppression by (**1**) time interval after PL enrollment date and (**2**) PL usage group

After a six-month run-in period, we evaluated viral suppression status in separate 6-month increments following a member’s enrollment date. We found a significantly larger proportion of individuals in the high usage group maintained viral suppression during the entire 6–12-month period following enrollment relative to the low usage group (92.3% vs. 81.0% *p* = 0.008). The same was independently true for the 12–18-month period (92.9% vs. 80.0% *p* = 0.020) (Fig. 3). For subsequent 6-month intervals, the percentage of members in the high usage group that were suppressed continued to remain greater than 90%--though the number of people with sufficient data significantly diminished in subsequent time intervals. In contrast, the proportion of those suppressed in the comparator low usage group remained at least 10% less than the high usage group.

### Stress Scores Preceding Loss of Viral Load Suppression

**Fig. 4:**
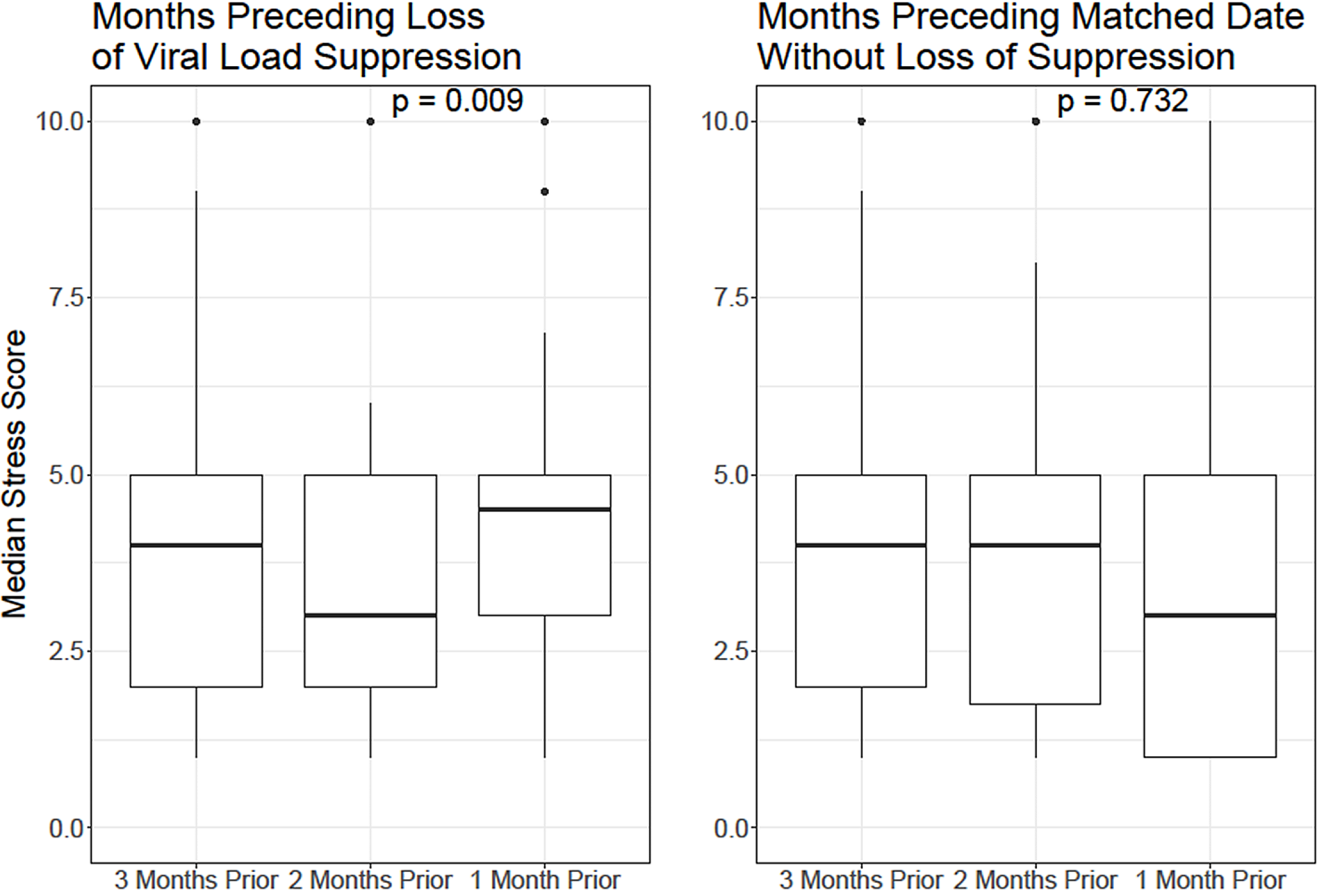
Median stress scores preceding first loss of viral load suppression and preceding a comparable date among members who remained suppressed

After a similar 6-month run-in period, there were 122 confirmed events of viral loads becoming unsuppressed from suppressed in individuals who had non-missing stress score data prior to the event. After including only the first event per person, there were 66 unique members and events remaining (Fig. 4). The median stress score in the one month preceding a member’s first occurrence of viral load becoming unsuppressed was 4.5 (IQR, 3–5) (higher scores indicate greater stress) whereas the median stress score two and three months preceding viral load conversion was 3 (IQR, 2–5) and 4 (IQR, 2–5), respectively (*p* = 0.009), indicating greatest stress in the one month preceding loss of viral suppression. The comparator group without any loss of VLS did not have a significant difference in stress score distribution when comparing the three months preceding the comparable event date (*p* = 0.732).

## Discussion

To our knowledge, this is the first analysis to describe long-term use of an mHealth app to support engagement with HIV care and associated clinical outcomes. PL, deployed in 2017 as usual care in a moderately sized RWHAP-funded non-urban clinic, has accrued a large cohort of PL participants who have accumulated more than 10,101 person-months of engagement with PL in this time period. High users of PL made up over 80% of the cohort in every 6-month interval after enrollment regardless of viral suppression status. Usage of the app may have been improved for all users by incorporating the telehealth feature prior to the pandemic, but independent of this over 90% of those high users had consistent viral suppression. In multi-variable Cox regression model, high use of PL is independently associated with higher probability of sustained viral suppression over the entire period of observation. While high PL use is independently associated with sustained viral suppression, other factors are also associated with greater likelihood of maintaining viral suppression, including older age and higher income when controlling for other factors. These same factors are associated with higher app use. Over two-thirds of all members who were unsuppressed prior to their enrollment in PL became suppressed for their entire enrollment period, suggesting significant benefit of this intervention over a sustained period of time.

We found that among PL users who were virally suppressed at baseline, 25% of individuals with low PL usage developed an unsuppressed viral load by month 18, and 50% of individuals with low PL usage developed an unsuppressed viral load by month 30. In contrast, high users of PL did not exceed a 25% probability of developing an unsuppressed viral load even after 36 months of follow-up. These results build upon findings in a 24-month follow-up of PL members which found that there were more high PL users with viral suppression over time than low PL users [19], and an earlier 12-month follow-up study [18]. Both of those earlier studies were limited by lower sample sizes. In the current study, we provide results with a larger sample size for up to 36 months by replicating and extending previous methods and results.

In addition, in an exploratory analysis, we identified a significant increase in self-reported stress scores in the month prior to loss of VLS compared with the previous months. To reduce confounding, we compared these scores with similarly timed stress scores among people who did not lose viral load suppression, and we found no significant increase in stress levels among this comparator group. These findings suggest that higher self-reported stress increases the risk of losing viral load suppression. This finding provides an important foundation for future studies seeking to identify early warning signs of an impending loss of viral load suppression.

### Limitations

This study was a retrospective cohort evaluation of PL as usual care, which conveys the limitations of lack of randomization and a reduction in sample size over time due to varying enrollment dates with a fixed end date of monitoring. This may decrease the level of confidence in the later observations which are truncated in time. Though smartphone reminders appear without prompting, PL is reliant on self-monitoring of one’s own behaviors and may require patient insight into personal motivations, which is similar to other mHealth applications [30–33]. Because the end user must “apply” knowledge gleaned from the app to improve health behaviors, low literacy related to health, technology, or language may act as potential barriers to app use and may intersect with other barriers to achieving and maintaining viral suppression. Furthermore, without sample randomization, our results may be biased towards those who use the app more, because they may also be more engaged in their own healthcare at baseline. One clinically meaningful way of addressing this discrepancy among those with lower income will be to invest in targeted removal of barriers such as investing further in provision of reliable access to smart phones, technology education, multilingual accommodations, and more icon or picture-based features rather than relying on written language comprehension. Lastly, in our overall analysis of factors associated with an unsuppressed viral load, we calculated PL usage based on activity during a member’s entire enrollment period to maintain consistency in comparing people who did and did not have loss of viral load suppression. This will slightly limit generalizability of our conclusions but was mitigated in the survival analysis by measuring app usage solely prior to the first loss of viral suppression.

In the face of these limitations, we note that while PL may be one important intervention in maintaining viral suppression over time, it is not sufficient to address every barrier our patients face—especially among younger adults and those with lower incomes. There is still a need for development of tailored programs for these groups. In the current study, low use of the app was associated with significantly higher probability of viral rebound within the first year of observation. In addition, rates of viral non-suppression increased over each six-month increment in low app users. It may be possible to develop algorithms to identify and signal specific patterns of reporting or app use that are predictive of developing viral non-suppression—such as overall disengagement with check-ins or a sudden increase in self-reported stress scores for an individual user. An early warning system based on an increase in self-reported stress may provide a critical opportunity for earlier outreach by support staff to mitigate factors that may lead to worse health outcomes. Maintaining this remote connection may also play a particularly important role for those physically disconnected from care, including rural populations, urban populations who lack transportation, or when disruptions to in-person care due to global events such as the Covid-19 pandemic cause further isolation.
